# New metabolic health definition might not be a reliable predictor for mortality in the nonobese Chinese population

**DOI:** 10.1186/s12889-022-14062-3

**Published:** 2022-08-29

**Authors:** Ziqiong Wang, Yan He, Liying Li, Muxin Zhang, Haiyan Ruan, Ye Zhu, Xin Wei, Jiafu Wei, Xiaoping Chen, Sen He

**Affiliations:** 1 Department of Cardiology, West China Hospital of Sichuan University, Chengdu, 610041 China; 2 Department of Interventional Operating Room, Mianyang People’s Hospital, Mianyang, China; 3 Department of Cardiology, First People’s Hospital, Longquanyi District, Chengdu, China; 4 Department of Cardiology, Hospital of Traditional Chinese Medicine, Shuangliu District, Chengdu, China; 5 Department of Cardiology and National Clinical Research Center for Geriatrics, West China Hospital of Sichuan University, Chengdu, China

**Keywords:** All-cause mortality, Metabolic health, Metabolically unhealthy, Non-obese individuals

## Abstract

**Background:**

Recently, a new metabolic health (MH) definition was developed from NHANES-III. In the origin study, the definition may stratify mortality risks in people who are overweight or normal weight. We aimed to investigate the association between the new MH definition and all-cause mortality in a nonobese Chinese population.

**Methods:**

The data were collected in 1992 and then again in 2007 from the same group of 1157 participants. The association between the new MH definition and all-cause mortality were analyzed by Cox regression models with overlap weighting according to propensity score (PS) as primary analysis.

**Results:**

At baseline in 1992, 920 (79.5%) participants were categorized as MH, and 237 (20.5%) participants were categorized as metabolically unhealthy (MUH) based on this new definition. During a median follow-up of 15 years, all-cause mortality occurred in 17 (1.85%) participants in MH group and 13 (5.49%) in MUH group, respectively. In the crude sample, Kaplan–Meier analysis demonstrated a significantly higher all-cause mortality in MUH group when compared to MH group (log-rank *p* = 0.002), and MUH was significantly associated with increased all-cause mortality when compared to MH with HR at 3.04 (95% CI: 1.47–6.25, *p* = 0.003). However, Kaplan–Meier analysis with overlap weighting showed that the cumulative incidence of all-cause mortality was not significantly different between MH and MUH groups (adjusted *p* = 0.589). Furthermore, in the primary multivariable Cox analysis with overlap weighting, adjusted HR for all-cause mortality was 1.42 (95% CI: 0.49—4.17, *p* = 0.519) in MUH group in reference to MH group. Other additional PS analyses also showed the incidence of all-cause mortality was not significantly different between the two groups.

**Conclusion:**

The new MH definition may be not appropriate for mortality risk stratification in non-obese Chinese people. Further investigations are needed.

**Supplementary Information:**

The online version contains supplementary material available at 10.1186/s12889-022-14062-3.

## Introduction

Metabolic abnormalities are often observed in obesity, but it is not always true. Among the obese individuals, not all subjects present metabolic abnormalities, namely the metabolically healthy obesity (MHO) phenotype [1]. For nonobese individuals, some of them can exhibit abnormal metabolic profiles, namely the metabolically unhealthy non-obese phenotype (MUNO) [2]. It is well known that the unhealthy metabolic status, when compared to the obesity per se, has played a more important role in the development of cardiovascular diseases and type 2 diabetes, and thus resulting in a higher mortality risk. In previous studies, the absence of metabolic syndrome and its components or absence of insulin resistance were widely used to define metabolic health (MH) [3, 4]. However, there is still certain insufficiency of those previous definitions and criteria to identify individuals with truly MH [5–8]. Recently, a new definition of MH has been proposed by Zembic et al. based on the data from the third National Health and Nutrition Examination Survey (NHANES-III) and validated in UK biobank cohort [9]. It was shown that participants categorized as MHO by this new definition were not at increased risk for cardiovascular disease and total mortality, while participants categorized as metabolically unhealthy (MUH) have a substantially higher risk. In addition, the risks of aforementioned outcomes were almost equally increased in participants with metabolically unhealthy normal weight and metabolically unhealthy obesity, indicating the new MH definition may also help to stratify mortality risk in non-obese individuals.

To some extent, nonobese individuals have not been focused with regards to the prevention of cardiometabolic diseases, which are more commonly related to obesity. According to previous data, the prevalence of metabolically unhealthy normal weight phenotype is 10–37% based on the different ethnic population examined [10]. What’s more, some studies have shown that Asians are more likely to be MUNO than typically obese [11]. This phenotype is characterized by a higher content of visceral adipose tissue and fat mass, reduced insulin sensitivity, and dyslipidemia [2]. It was demonstrated that individuals with MUNO or metabolically obese normal-weight (MONW) were at higher risk of increased arterial stiffness and carotid atherosclerosis [12], stroke [13, 14], as well as higher risk of all-cause mortality and cardiovascular mortality [15, 16] when compared to MHO. The risk for all-cause mortality and/or cardiovascular events could be more than threefold higher in metabolically unhealthy individuals with normal wight than that in metabolically healthy individuals with normal weight [2]. Those findings highlighted that it maybe the abnormal metabolic profile, rather than obesity defined by BMI, placing individuals at increased risk for cardiovascular diseases and mortality. Therefore, identification of non-obese individuals at high risk is important and meaningful. What’s more important is that not just screening people by some anthropometric parameters (e.g., BMI), but also valuing the metabolic markers, or combining the two aspects together. In this study, we aimed to investigate the clinical significance of the new defined MH for all-cause mortality in a nonobese Chinese population. Meanwhile, the new defined MH could be associated with some other variables, which may mediate or suppress the relationship between MH and mortality. Therefore, we also investigated whether other variables mediated the relationship between the new defined MH and mortality.

## Participants and methods

### Study population

**Fig. 1:**
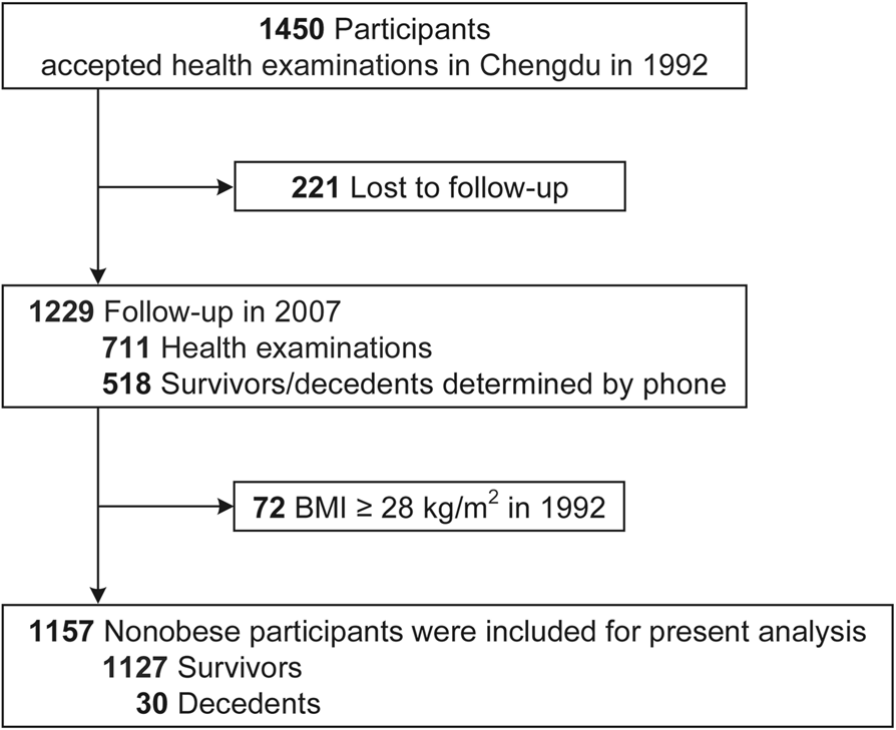
Flow diagram

The present study used a subset of participants from the Chinese Multi-Provincial Cohort Study [17, 18]. A stratified random sampling for each sex and 10-year age group was performed. Overall, in 1992, a group of 1450 individuals aged 35–64 years received health survey in an urban community of Chengdu, Sichuan province, China. In 2007, we conducted another health survey on the same group of participants. The two surveys were supported by a project from the National Eighth Five-Year Research Plan and megaprojects of science research for China's 11th 5-year plan, respectively. Among the 1450 individuals, 711 individuals received an interview health survey in 2007, and telephone follow-ups were conducted for the remaining individuals (*n* = 518). After excluding the individuals who were lost to follow-up and the obese individuals (body mass index, BMI ≥ 28 kg/m^2^) [19], a total of 1157 nonobese participants with complete data were analyzed (Fig. 1). Other detailed information of these participants has been reported elsewhere [17, 18, 20].

The surveys were approved by the Ministry of Health of China, as well as by the Ethics Committee of West China Hospital of Sichuan University. The study protocol conforms to the ethical guidelines of the Declaration of Helsinki. All participants provided written informed consent.

### Data collection

At baseline in 1992, the survey content included standardized questionnaire, anthropometric measurements, and laboratory tests. Standardized questionnaire collected the information on demographic characteristics, such as age, sex, etc. Based on the standard methods [21], we performed anthropometric measurements, which included blood pressure, height, weight, waist circumference, hip circumference. Laboratory tests consisted of fasting plasma glucose (FPG) and fasting lipid profiles, including triglycerides, total cholesterol, high density lipoprotein-cholesterol (HDL-C), and low-density lipoprotein cholesterol (LDL-C).

### Related definitions

According to the original study, the criteria for the new MH definition are as follows: 1) systolic blood pressure (SBP) less than 130 mmHg and no use of blood pressure-lowering medication, 2) waist to hip ratio (WHR) less than 0.95 for women and less than 1.03 for men, 3) no prevalent diabetes [9]. Individuals who met all the criteria were categorized as MH, otherwise, were categorized as MUH.

Other definitions used in the study were as follows. Cardiovascular diseases were defined as self-reported coronary heart disease and/or cerebral stroke. Diabetes was defined by self-reported history or FPG ≥ 7.0 mmol/L. WHR was calculated as follows: WHR = waist circumference/hip circumference. BMI was calculated as follows: BMI = Weight (Kg)/Height^2^ (m^2^). Smoking was categorized as never, current, and past. Alcohol intake was defined as average intake of alcohol ≥ 50 g/day. Physical activity was defined as exercise one or more times per week, at least 20 min for each time [17, 18, 20].

### Endpoint

The primary end point was all-cause mortality from study baseline in 1992 to follow-up in 2007. The occurrence of all-cause mortality and the cause of mortality was confirmed via telephone contact with referring relatives.

### Statistical analysis

For summarizing baseline characteristics of subjects, continuous variables were presented as mean ± standard deviation (SD) and median with interquartile range (IQR) where appropriate, and categorical variables as number (percentage) for each group. Comparisons of baseline characteristics between subjects who finished follow-up and those who lost to follow-up were performed using the analysis of variance or Kruskal–Wallis tests for continuous variables, and the chi-square or Fisher exact tests for categorical variables.

Given the observational nature of the present study, propensity scores (PS) were developed to account for potential confounding by observed baseline characteristics. PS methods replace an entire set of baseline characteristics with a single composite score, and this can be accomplished with a number of potential confounders in excess of what is possible with conventional regression methods [22, 23]. The individual propensities for diagnosis of MH were estimated with the use of a multivariable logistic-regression model that included the following covariates, including age, sex, smoking, drinking, exercise, cardiovascular diseases, diastolic blood pressure (DBP), total cholesterol, HDL-C, LDL-C, triglycerides, and BMI. Then, associations between MUH and all-cause mortality were estimated by Cox regression models with the use of three PS methods, including overlap weighting, propensity-score matching (PSM), and the PS as an additional covariate. Direct acyclic graph was built to select variables for adjustment in multivariable Cox proportional regression models.

Overlap weighting was chosen as the primary method for confounder adjustment in this study, because it could minimize the influence of extreme PS on model output [24]. Overlap weighting could assign weights to each patient that are proportional to the probability of that patient belonging to the opposite exposed group. Specifically, exposed participants are weighted by the unexposed probability (1 – PS), and unexposed participants are weighted by the exposed probability (PS). Overlap weighting assigns greater weight to participants in which treatment cannot be predicted and lesser weight to patients with extreme PS (approaching 0.0 or 1.0) preventing outliers from dominating the analysis and decreasing precision, which is a concern with inverse probability weighting [25]. Furthermore, overlap weighting has the favorable property of resulting in the exact balance (standardized mean differences [SMD] = 0) of all variables included in the multivariable logistic regression model used to derive the PS. PSM was also used to adjust for clinically relevant baseline characteristics that were potentially confounding variables, and participants were matched 1:1 using the nearest neighbor method, with a fixed caliper width of 0.08. After overlap weighting and PSM, SMD were estimated for the baseline covariates before and after the processes to assess pre-match imbalance and post-match balance, and absolute SMD of less than 0.1 for a given covariate indicate a relatively small imbalance [26]. In addition, cumulative hazard plots were also produced to display the cumulative incidence of all-cause mortality in different methods.

To estimate the plausibility of bias from unmeasured and residual confounding, we calculated E-values, which could assess the potential for unmeasured confounding between MUH and all-cause mortality, and it quantifies the required magnitude of an unmeasured confounder that could negate the observed association between MUH and all-cause mortality [27]. In addition, mediation analysis, a single mediator model, was also conducted to assess whether the relationship between MUH and all-cause mortality was mediated or suppressed by other variables. In these analyses, mortality status was used as the outcome variable. MUH was used as the predictor, and other variables were used as mediators, separately.

The statistical analyses were performed with R software, version 4.1.0 (R Project for Statistical Computing) mainly including the “MatchIt” [28], “survival” [29], “survey” [30], “cobalt” [31], “mediation” [32], and “Evalue” [33] packages. For all statistical analyses, a two-sided *p* value of 0.050 was considered statistically significant.

## Results

### Baseline characteristics in 1992

**Fig. 2:**
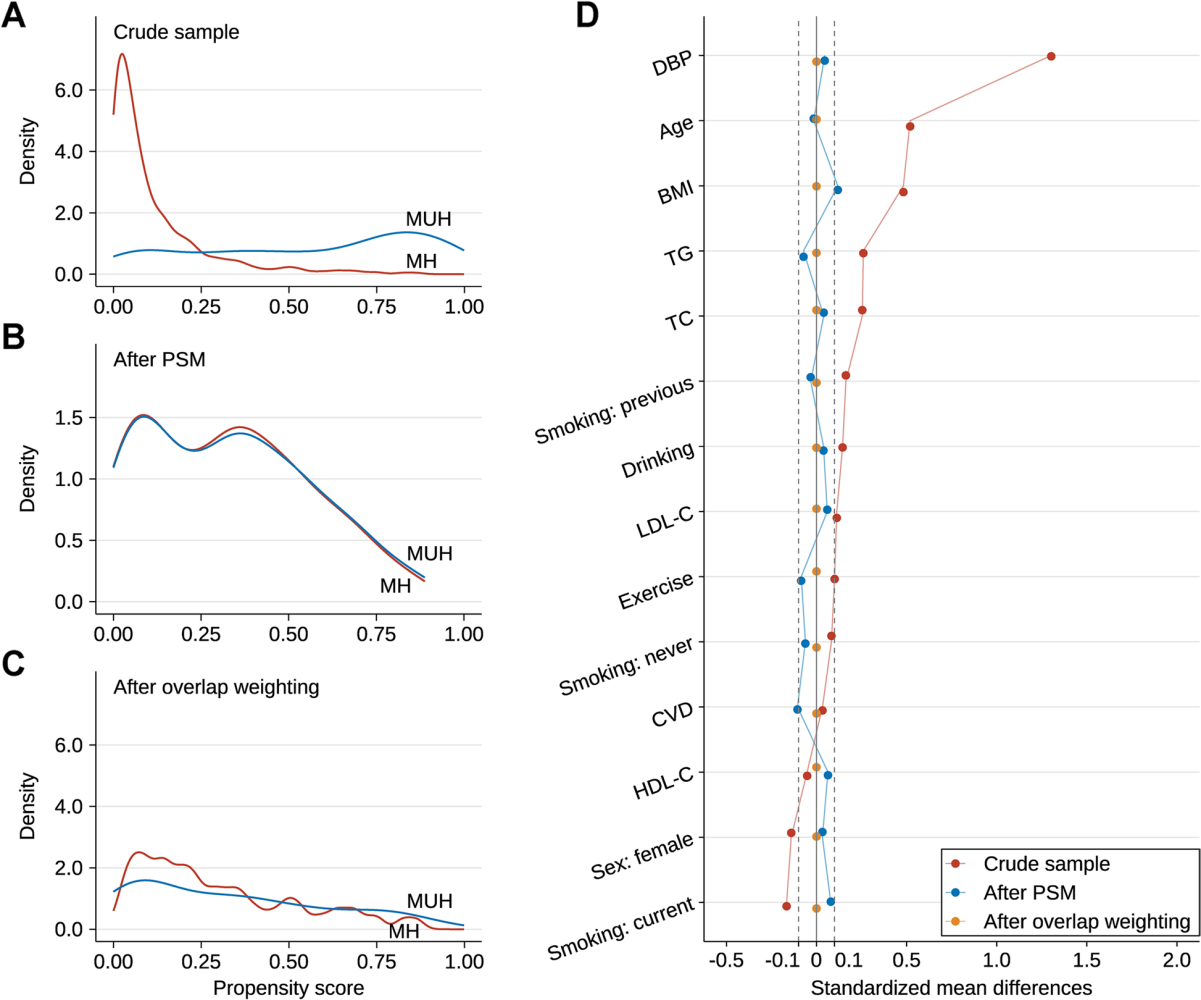
Propensity score distributional overlap and absolute standardized differences in the different groups based on the new MH definition in the crude cohort, PSM cohort, and overlap weighting cohort. **A**: PS distributions between MH and MUH groups in crude cohort. **B**: PS distributions between MH and MUH groups in PSM cohort. **C**: PS distributions between MH and MUH groups in overlap weighting cohort. **D**: standardized mean differences in the participants stratified by the new MH definition

In 1992, 1450 individuals accepted health examinations. Among them, 221 individuals were lost to follow-up. As shown in table S1, most of the baseline characteristics between individuals who finished follow-up and those who were lost to follow-up did not have significant differences except three variables, namely age, sex, and hip circumference. In total, 1157 nonobese subjects with complete data were included for the present analysis. Baseline characteristics for individuals with MH and with MUH before matching and after matching and after overlapping are shown in table S2. There were 920 individuals in MH group and 237 individuals in MUH group before matching. There were differences between the two groups in several of the baseline variables (Table S2 and Fig. 2D).

The β coefficients for predicting MUH according to all the variables included in PS model are presented in Table 1, and the C-statistic was 0.88. After matching, all SMDs were less than 0.100 for all variables except BMI, indicating only small imbalance between the two groups (Table S2 and Fig. 2D). After overlap weighting, SMDs for all characteristics were < 0.100, which also indicated that the weighted population in the two groups was subsequently comparable (Table S2 and Fig. 2D). As shown in Fig. 2A, prior to matching and overlap weighting, lesser overlap of PS curves of the two groups indicated a greater risk of confounding. After matching, PS curves for MH and MUH were superimposed, indicating that the baseline differences between the two groups were largely attenuated (Fig. 2B). After overlap weighting, the overall distribution of PS was balanced between MH and MUH (Fig. 2C).

**Table 1:** Beta coefficient of MUH for all variables included in the propensity score model

Variables	Changes	Beta	Std.error	*p* values
Intercept		-21.154	1.785	0.000
Sex	female vs male	-0.079	0.270	0.771
Age (years)	increase 1 unit	0.081	0.018	0.000
Smoking				
never		ref		
previous		0.561	0.533	0.293
current		-0.355	0.258	0.169
Drinking	yes vs no	0.242	0.280	0.387
Exercise	yes vs no	0.552	0.231	0.017
Cardiovascular diseases	yes vs no	1.006	0.718	0.161
DBP (mmHg)	increase 1 unit	0.201	0.014	0.000
TC (mmol/L)	increase 1 unit	0.196	2.101	0.926
LDL-C (mmol/L)	increase 1 unit	-0.020	2.098	0.992
HDL-C (mmol/L)	increase 1 unit	-0.954	2.109	0.651
Triglycerides (mmol/L)	increase 1 unit	0.098	0.971	0.920
BMI (kg/m^2)	increase 1 unit	0.020	0.043	0.647

*Abbreviations*: *MUH* Metabolically unhealthy, *DBP* Diastolic blood pressure, *TC* Total cholesterol, *LDL-C* Low density lipoprotein cholesterol, *HDL-C* High density lipoprotein cholesterol, *BMI* Body mass index

C-index = 0.880

### Endpoint

During a follow-up of 15 years, all-cause mortality occurred in 30 participants. Among them, there were 5 cancer related death, and 2 stroke related death. The cause of deaths could not be confirmed in 23 participants. The all-cause mortality rate was 1.85% (*n* = 17) in MH group and 5.49% (*n* = 13) in MUH group, respectively.

### Survival analysis

**Fig. 3:**
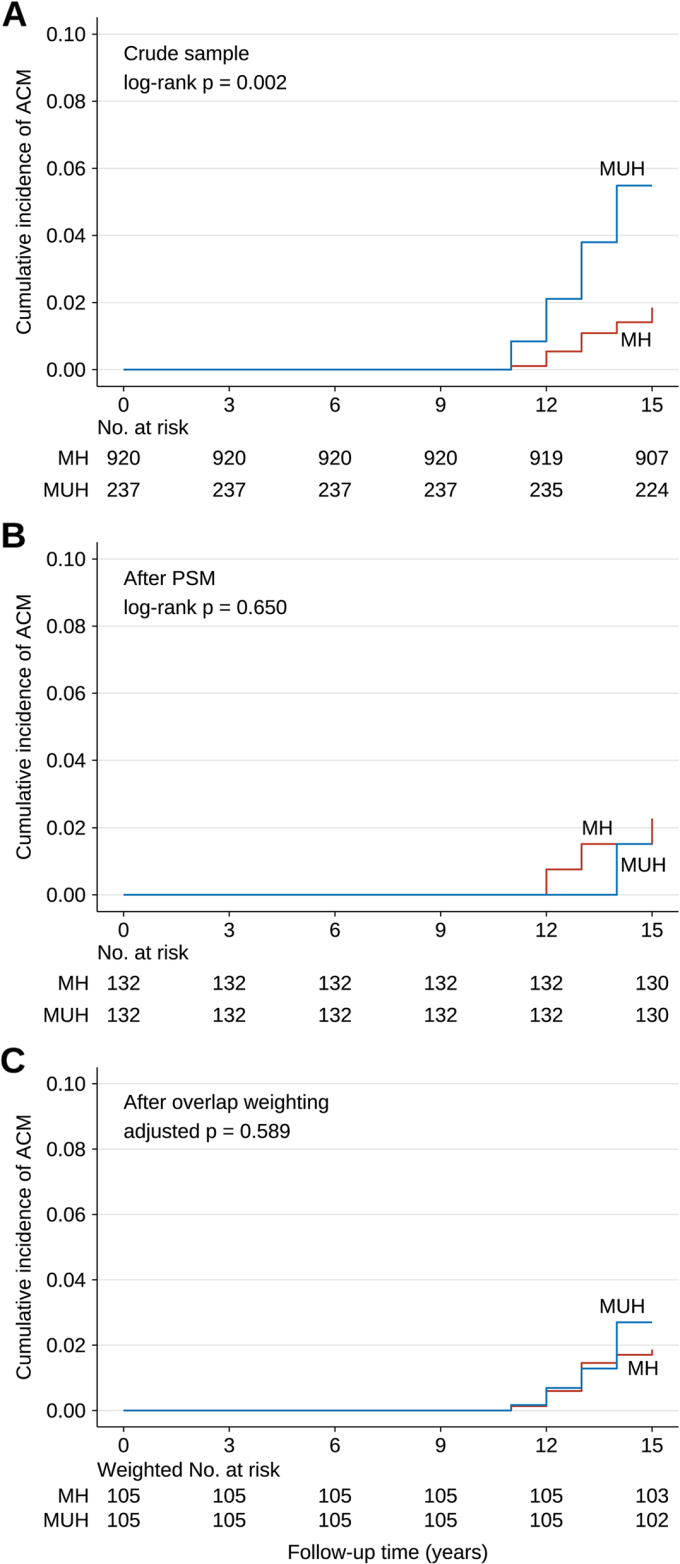
Kaplan–Meier (KM) survival curves for all-cause mortality (MUH vs. MH). **A** KM curves in the crude sample; **B** KM curves in the PSM sample; **C** KM curves in the overlap weighting sample

Figure 3A depicts the Kaplan–Meier curves for all-cause mortality in the crude sample, and the cumulative incidence of all-cause mortality is significantly higher in participants with MUH when compared to those with MH (log-rank *p* = 0.002). In the crude analysis, individuals with MUH were more likely to have died (the primary endpoint) than those with MH (HR: 3.04, 95% CI: 1.47–6.25, *p* = 0.003) (Table 2). After adjusting for potential confounding factors, including age, sex, smoking, drinking, exercise, cardiovascular diseases, DBP, total cholesterol, HDL-C, triglycerides, LDL-C, and BMI based on the results of direct acyclic graph (Figure S1), HR was 1.09 (95% CI: 0.38–3.13, *p* = 0.875). For including many covariates, the convergence of the model may be poor, and the results were exploratory.

**Table 2:** Associations between MUH and all-cause mortality in the crude analysis, multivariable analysis and propensity-score analyses

Analysis	All-cause mortality
No. of deaths/no. of participants at risk (%)^a^	
MH	17/920 (1.85%)
MUH	13/237 (5.49%)
Crude analysis	3.04 (1.47, 6.25), 0.003
Propensity-score analyses	
With overlap weighting (univariable)	1.45 (0.50, 4.19), 0.490
With overlap weighting (multivariable)^b^	1.42 (0.49, 4.17), 0.519
With PSM (univariable)	0.66 (0.11, 3.97), 0.652
With PSM (multivariable)^c^	0.69 (0.11, 4.20), 0.685
Adjusted for PS^d^	1.18 (0.39, 3.61), 0.766
Adjusted for PS^e^	1.16 (0.39, 3.45), 0.790
Multivariable analysis^e^	1.09 (0.38, 3.13), 0.875

Values are n (%) or HRs (95% CI) with *p* values

^a^Binary event rates

For the relatively small number of all-cause mortality, multivariable models only adjusted for some basic variables to ensure the convergence of the model: ^b^adjustment for age and sex; ^c^adjustment for age, sex, DBP, TC and HDL-C; ^d^adjustment for propensity score; ^e^adjustment for propensity score plus age and sex. ^f^Adjustment for age, sex, smoking, drinking, exercise, cardiovascular diseases, DBP, TC, HDL-C, triglycerides, LDL-C and BMI; for including many covariates, the convergence of the model may be poor, and the results were exploratory

*Abbreviations*: *MH* Metabolic health, *MUH* Metabolically unhealthy, *PSM* Propensity score matching, *PS* Propensity score

No significant difference in cumulative all-cause mortality was observed between MH and MUH subgroups in PSM cohort (log-rank *p* = 0.650) (Fig. 3B). Both univariable (HR: 0.66, 95% CI: 0.11–3.97, *p* = 0.652) and multivariable (HR: 0.69, 95% CI: 0.11–4.20, *p* = 0.685) PSM Cox models showed that MUH was not significantly associated with increased mortality (Table 2).

Overlap weighting-adjusted Kaplan–Meier analysis also showed that the cumulative incidence of all-cause mortality is not significantly different between participants with MH or MUH (log-rank *p* = 0.589) (Fig. 3C). In the primary univariable and multivariable Cox regression analysis with overlap weighting, no significant association between MUH and all-cause mortality was revealed. The HRs were 1.45 (95% CI: 0.50–4.19, *p* = 0.490) and 1.42 (95% CI: 0.49–4.17, *p* = 0.519), respectively (Table 2). In the last, after including PS as another covariate, the results remained the same with HR at 1.18 (95% CI: 0.39–3.61, *p* = 0.766) and 1.16 (95% CI: 0.39–3.45, *p* = 0.790) in the univariable and multivariable analyses, respectively (Table 2).

### Sensitivity analysis

In weighted multivariable Cox proportional hazards model, the E-value for this point estimate is 2.19 and for the upper confidence interval limit is 7.81. This result suggested that only when an unmeasured confounder existed with a higher relative risk for both MUH and all-cause mortality than the above-mentioned E-value could modify the conclusion that MUH was not associated with increased all-cause mortality as observed in this study. The results for mediation analysis for all-cause mortality are shown in Table S3. Within those mediation models, all total effect, direct effect of MUH on all-cause mortality, and indirect effect by other variables (e.g. age, sex, smoking, drinking, etc.) were consistently insignificant, which suggested that the insignificant association between MUH and all-cause mortality was not suppressed by other variables.

## Discussion

In this analysis involving a nonobese Chinese population, the risk of all-cause mortality was not significantly different among individuals who were classified as MH and MUH by this new definition. Based on the weighted multivariable Cox model, the E-value for the effect of the new MH definition on all-cause mortality was 2.19 in MUH versus MH. Further mediation analysis suggested that the effect of MUH on all-cause mortality was not mediated by other variables. The results indicated that this new MH definition might not be suitable for mortality risk stratification for nonobese Chinese people.

A meta-analysis showed that the prevalence of MONW around the world varies largely, ranging from 6.6% to 45.9% [34]. This heterogeneity was affected by several factors, including participants’ age, gender, ethnicities, region, sample size, MONW criteria (criteria for obesity and MH), and so on. In a recent study, Zheng et al. demonstrated that the overall prevalence of MONW was 16.1% in a general Chinese population. Individuals were considered as MONW if they had at least two metabolically abnormal trait based on the metabolic syndrome criteria from the International Diabetes Federation in 2015 and BMI of 18.5–23.9 kg/m^2^ in this study [35]. While in a more previous study, Zhang et al. reported that the prevalence of MONW was as low as 4.3% in a Chinese Beijing urban cohort. In this study, MONW was defined as BMI of 18.5–25 kg/m2 and metabolic abnormality referenced at least 3 abnormal traits among the factors of blood pressure, waist circumference, triglycerides, FPG, and HDL-C. [36]. In our present study, according to the new MH definition, the prevalence of MUNO was 20.5%. As we can see, there are various criteria to evaluate MUNO/MONW currently, no consensus has been reached to a final definition, and thus interpretation of those results, or comparisons of prevalence across different studies should be cautious.

In the univariable analysis for the crude sample, MUH defined by the new defined MH was a significantly risk factor for all-cause mortality in our nonobese participants. However, after adjustment for potential confounders and PSM, the association changed materially. The mixed results between the original study and the present study might be explained by several reasons. Firstly, different BMI categories and cutoffs. In the original study, there were three BMI categories, namely normal weight (BMI, 18.5–24.9 kg/m^2^), overweight (BMI, 25.0–29.9 kg/m^2^), and obesity (BMI, ≥ 30 kg/m^2^). In our study, the participants were all non-obese with BMI less than 28 kg/m^2^. In addition, the cutoff value of WHR may also not be optimal for Chinese people due to the different ethnicities and baseline characteristics. Secondly, the new MH definition only took SBP into consideration but not DBP since it failed to achieve statistical significance to predict outcomes in the original study. In our study, DBP is also a significant risk factor for all-cause mortality. Historical studies have revealed a J-curve relation between DBP and cardiovascular outcomes [37], as well as cardiovascular and all-cause death [38]. In this case, higher DBP could also potentially lead to adverse prognosis. Thirdly, comparing to traditional metabolic criteria, the biggest distinctions for the new MH definition is the lack of dyslipidemia, which is also a well-established risk factor for cardiovascular diseases and mortality [39, 40].

Due to the relatively small sample size, we constructed several models to illustrate the association between MUH and all-cause mortality. Although adjustment attenuated the crude effect of MUH and all-cause mortality, point estimates remained clinically significant for most of the analyses. As shown in Table 2, most of the effect sizes indicated that individuals with MUH defined by the new MH definition tended to have a higher risk of all-cause mortality. In the primary analysis with overlap weighting, individuals classified as MUH were at more than 40% higher risk of all-cause mortality when compared to those classified as MH, even if the results did not achieve statistically significance. We further conducted mediation analysis to examine whether the insignificant association between MUH and all-cause mortality was suppressed by other mediators. As shown in table S3, the results indicated that there was no suppressing effect. It seems that the new MH definition could not be able to stratify mortality risk in the non-obese Chinese group. However, statistical insignificance does not automatically equate to a unmeaningful or impractical effect [41, 42]. Especially considering the small sample size, with only 1157 participants and 30 all-cause mortality in the present study.

To our knowledge, this is the first study to assess the role of the new MH definition for all-cause mortality in a non-obese Asian population. The negative results based on multiple statistical analyses in the present study indicated that the generalization of the new definition in other populations needs to be validated. However, this study has several limitations. Firstly, the mortality was relatively low in our study. For the relatively small number of all-cause mortalities, multivariable models only adjusted for some basic variables to ensure the convergence of the model. On the other hand, for including those covariates, the convergence of the model may be poor, and the results were exploratory. Secondly, most of the specific cause for death could not be determined. We can only make a conclusion about the relationship between the new MH definition and all-cause mortality. Third, the relatively small sample from a single center might also affect the statistical power of the results. Therefore, the explanation of the current study needs to be cautious because of those limitations. Multicenter-based larger studies are needed to confirm and extend the present finding.

## Conclusions

In this study, we firstly assessed the performance of the new MH definition, based on SBP, use of BP medication, WHR and self-reported diabetes, for all-cause mortality in a non-obese Chinese population. Our results suggested that the risk of total mortality was not significantly different between the non-obese people with MH or MUH classified by this definition. This new MH definition may not be suitable for mortality risk stratification for non-obese Chinese people. Further studies are needed to explore the role of this new MH definition in different populations.

## Supplementary Information

**Additional file 1: Figure S1.** Direct acyclic graph: risk factors and all-cause mortality. Abbreviations: MUH = metabolically unhealthy, ACM = all-cause mortality, CVD = cardiovascular diseases. DBP = diastolic blood pressure, TC: total cholesterol, LDL-C: low density lipoprotein cholesterol, HDL-C = high density lipoprotein cholesterol, TG: triglycerides, BMI: body mass index.

**Additional file 2: Table S1.** Baseline characteristics of the individuals between follow-up and lost to follow-up.

**Additional file 3: Table S2.** Baseline characteristics of study cohort in 1992.

**Additional file 4: Table S3.** Mediation analysis (single mediator model).

## Data Availability

The datasets used and/or analyzed during the current study are available from the corresponding author on reasonable request.

## Abbreviations

MHO: Metabolically healthy obesity
MUNO: Metabolically unhealthy non-obese
MH: Metabolic health
NHANES-III: National Health and Nutrition Examination Survey-III
MUH: Metabolically unhealthy
MONW: Metabolically obese normal-weight
FPG: Fasting plasma glucose
HDL-C: High density lipoprotein-cholesterol
LDL-C: Low density lipoprotein cholesterol
SBP: Systolic blood pressure
WHR: Waist to hip circumference ratio
BMI: Body mass index
PS: Propensity score
DBP: Diastolic blood pressure
PSM: Propensity-score matching
