# Supplementary figures and images for: New metabolic health definition might not be a reliable predictor for mortality in the nonobese Chinese population
Source: BMC Public Health. 2022 Aug 29;22:1629. doi: 10.1186/s12889-022-14062-3 (PMC9422146; doi:10.1186/s12889-022-14062-3)

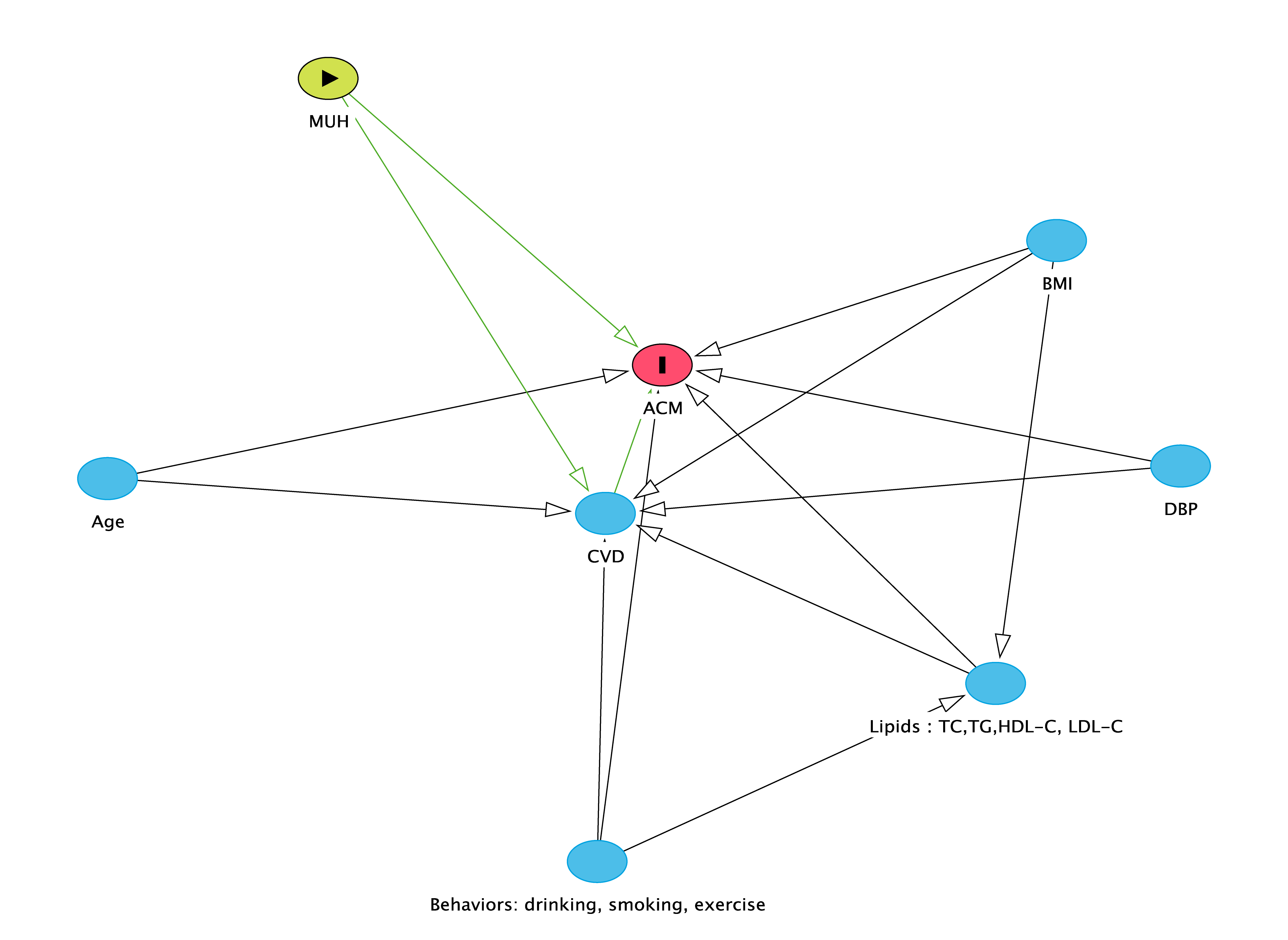

Supplement: Supplementary file 1 — Additional file 1: Figure S1. Direct acyclic graph: risk factors and all-cause mortality. Abbreviations: MUH = metabolically unhealthy, ACM = all-cause mortality, CVD = cardiovascular diseases. DBP = diastolic blood pressure, TC: total cholesterol, LDL-C: low density lipoprotein cholesterol, HDL-C = high density lipoprotein cholesterol, TG: triglycerides, BMI: body mass index. [file 12889_2022_14062_MOESM1_ESM.tif]
