# Supplementary material for: New metabolic health definition might not be a reliable predictor for mortality in the nonobese Chinese population
Source: BMC Public Health. 2022 Aug 29;22:1629. doi: 10.1186/s12889-022-14062-3 (PMC9422146; doi:10.1186/s12889-022-14062-3)
Supplement: Supplementary file 2 — Additional file 2: Table S1. Baseline characteristics of the individuals between follow-up and lost to follow-up. [file 12889_2022_14062_MOESM2_ESM.docx]

| Table S1. Baseline characteristics of the individuals between follow-up and lost to follow-up. | | | |
| --- | --- | --- | --- |
|  | Follow-up (n = 1229) | Lost to follow-up (n = 221) | p values |
| Sex (female) | 449 (36.5) | 63 (28.5) | 0.026 |
| Age (years) | 48.00 (44.00, 53.00) | 49.00 (45.00, 54.00) | 0.015 |
| Smoking |  |  | 0.250 |
| never | 689 (56.1) | 111 (50.2) |  |
| previous | 38 (3.1) | 9 (4.1) |  |
| current | 502 (40.8) | 101 (45.7) |  |
| Drinking | 172 (14.0) | 28 (12.7) | 0.674 |
| Exercise | 292 (23.8) | 44 (19.9) | 0.245 |
| Cardiovascular diseases | 21 (1.7) | 4 (1.8) | 1.000 |
| DBP (mmHg) | 72.00 (70.00, 80.00) | 71.00 (70.00, 78.00) | 0.265 |
| TC (mmol/L) | 4.50 (3.90, 5.00) | 4.30 (3.90, 5.00) | 0.502 |
| LDL-C (mmol/L) | 2.20 (1.70, 2.70) | 2.20 (1.70, 2.70) | 0.825 |
| HDL-C (mmol/L) | 1.30 (1.10, 1.40) | 1.20 (1.10, 1.40) | 0.774 |
| Triglycerides (mmol/L) | 1.90 (1.50, 2.50) | 1.80 (1.50, 2.40) | 0.419 |
| BMI (kg/m^2) | 23.10 (21.40, 25.20) | 22.70 (20.80, 25.00) | 0.085 |
| Elements of new MH definition |  |  |  |
| SBP (mmHg) | 110.00 (104.00, 120.00) | 110.00 (104.00, 120.00) | 0.804 |
| waist (cm) | 76.00 (71.00, 82.00) | 75.00 (70.00, 81.00) | 0.482 |
| hip (cm) | 92.00 (88.00, 95.00) | 90.00 (86.00, 95.00) | 0.038 |
| FPG (mmol/L) | 4.20 (3.80, 4.90) | 4.20 (3.80, 4.90) | 0.572 |

Abbreviations: DBP = diastolic blood pressure, TC = total cholesterol, LDL-C = low density lipoprotein cholesterol, HDL-C = high density lipoprotein cholesterol, BMI = body mass index, MH = metabolic health, SBP = systolic blood pressure, FPG = fasting plasma glucose.
