# Supplementary material for: New metabolic health definition might not be a reliable predictor for mortality in the nonobese Chinese population
Source: BMC Public Health. 2022 Aug 29;22:1629. doi: 10.1186/s12889-022-14062-3 (PMC9422146; doi:10.1186/s12889-022-14062-3)
Supplement: Supplementary file 3 — Additional file 3: Table S2. Baseline characteristics of study cohort in 1992. [file 12889_2022_14062_MOESM3_ESM.docx]

| Table S2. Baseline characteristics of study cohort in 1992. | | | | | | | | | | | |
| --- | --- | --- | --- | --- | --- | --- | --- | --- | --- | --- | --- |
| Variables | Crude sample (n = 1157) | | |  | After PSM (n = 264) | | |  | After overlap weighting | | |
|  | MUH (n = 237) | MH (n = 920) | SMD |  | MUH (n = 132) | MH (n = 132) | SMD |  | MUH | MH | SMD |
| Sex (female) | 73 (30.8) | 343 (37.3) | -0.140 |  | 41 (31.1) | 39 (29.5) | 0.033 |  | 33.7 (32.1) | 33.7 (32.1) | <0.001 |
| Age (years) | 51.00 (47.00, 55.00) | 48.00 (43.00, 52.00) | 0.519 |  | 49.00 (46.00, 54.00) | 50.00 (46.75, 54.00) | -0.015 |  | 49.00 (45.40, 54.00) | 50.00 (46.00, 54.00) | <0.001 |
| Smoking |  |  |  |  |  |  |  |  |  |  | <0.001 |
| never | 140 (59.1) | 506 (55.0) | 0.083 |  | 68 (51.5) | 72 (54.5) | -0.062 |  | 57.4 (54.7) | 57.4 (54.7) | <0.001 |
| previous | 14 (5.9) | 19 (2.1) | 0.163 |  | 8 (6.1) | 9 (6.8) | -0.032 |  | 4.0 (3.8) | 4.0 (3.8) | <0.001 |
| current | 83 (35.0) | 395 (42.9) | -0.166 |  | 56 (42.4) | 51 (38.6) | 0.079 |  | 43.6 (41.5) | 43.6 (41.5) | <0.001 |
| Drinking | 44 (18.6) | 119 (12.9) | 0.145 |  | 25 (18.9) | 23 (17.4) | 0.039 |  | 18.1 (17.2) | 18.1 (17.2) | <0.001 |
| Exercise | 65 (27.4) | 211 (22.9) | 0.101 |  | 34 (25.8) | 39 (29.5) | -0.085 |  | 27.9 (26.6) | 27.9 (26.6) | <0.001 |
| Cardiovascular diseases | 5 (2.1) | 15 (1.6) | 0.033 |  | 3 (2.3) | 5 (3.8) | -0.105 |  | 2.9 (2.8) | 2.9 (2.8) | <0.001 |
| DBP (mmHg) | 86.00 (78.00, 90.00) | 70.00 (68.00, 75.00) | 1.303 |  | 80.00 (70.75, 80.00) | 80.00 (70.00, 80.00) | 0.046 |  | 79.08 (70.00, 80.61) | 78.00 (73.00, 80.00) | <0.001 |
| TC (mmol/L) | 4.60 (4.10, 5.10) | 4.30 (3.90, 4.80) | 0.254 |  | 4.50 (4.10, 5.00) | 4.50 (3.98, 5.00) | 0.040 |  | 4.50 (4.10, 5.00) | 4.50 (4.00, 5.00) | <0.001 |
| LDL-C (mmol/L) | 2.30 (1.70, 2.90) | 2.20 (1.70, 2.70) | 0.113 |  | 2.30 (1.67, 2.80) | 2.30 (1.70, 2.70) | 0.060 |  | 2.30 (1.70, 2.90) | 2.30 (1.70, 2.70) | <0.001 |
| HDL-C (mmol/L) | 1.30 (1.10, 1.50) | 1.30 (1.10, 1.40) | -0.052 |  | 1.20 (1.00, 1.40) | 1.20 (1.00, 1.40) | 0.064 |  | 1.20 (1.00, 1.40) | 1.20 (1.10, 1.40) | <0.001 |
| Triglycerides (mmol/L) | 2.10 (1.60, 2.50) | 1.80 (1.50, 2.40) | 0.260 |  | 2.00 (1.60, 2.70) | 2.00 (1.58, 2.70) | -0.072 |  | 2.00 (1.60, 2.50) | 2.00 (1.60, 2.60) | <0.001 |
| BMI (kg/m^2) | 23.90 (22.20, 25.80) | 22.70 (21.00, 24.60) | 0.482 |  | 23.70 (21.87, 25.80) | 23.65 (21.60, 25.10) | 0.118 |  | 23.47 (21.80, 25.70) | 23.60 (21.80, 25.10) | <0.001 |
| Elements of new MH definition |  |  |  |  |  |  |  |  |  |  |  |
| SBP (mmHg) | 135.00 (130.00, 146.00) | 110.00 (100.00, 118.00) | 1.903 |  |  |  |  |  |  |  |  |
| waist (cm) | 79.00 (73.00, 84.00) | 75.00 (70.00, 80.00) | 0.463 |  |  |  |  |  |  |  |  |
| hip (cm) | 93.00 (89.00, 96.00) | 91.00 (87.00, 94.00) | 0.325 |  |  |  |  |  |  |  |  |
| FPG (mmol/L) | 4.50 (4.00, 5.40) | 4.20 (3.80, 4.70) | 0.335 |  |  |  |  |  |  |  |  |
| Values are median (IQR) or n (%).  PSM = propensity score matching, MUH = metabolically unhealthy, MH = metabolic health, SMD = standardized mean difference, DBP = diastolic blood pressure, TC = total cholesterol, LDL-C = low density lipoprotein cholesterol, HDL-C = high density lipoprotein cholesterol, BMI = body mass index, SBP = systolic blood pressure, FPG= fasting plasma glucose. | | | | | | | | | | | |
