# Supplementary material for: New metabolic health definition might not be a reliable predictor for mortality in the nonobese Chinese population
Source: BMC Public Health. 2022 Aug 29;22:1629. doi: 10.1186/s12889-022-14062-3 (PMC9422146; doi:10.1186/s12889-022-14062-3)
Supplement: Supplementary file 4 — Additional file 4: Table S3. Mediation analysis (single mediator model). [file 12889_2022_14062_MOESM4_ESM.docx]

| \| Table S3. Mediation analysis (single mediator model). \| \| \| \| \| --- \| --- \| --- \| --- \| \| Mediators \| Total effect \| Directed effect \| Indirect effect \| \| Age \| -3.82 (-2.69, 4.86), 1.00 \| -3.27 (-2.84, 4.84), 0.99 \| -0.55 (-0.75, 0.71), 0.98 \| \| Sex \| -4.05 (-2.69, 4.86), 0.99 \| -3.69 (-2.72, 4.81), 1.00 \| -0.36 (-0.26, 0.22), 0.98 \| \| Smoking \| -3.95 (-2.69, 4.85), 0.99 \| -4.18 (-2.76, 4.84), 0.98 \| 0.23 (-0.22, 0.20), 0.97 \| \| Drinking \| -3.81 (-2.67, 5.03), 0.99 \| -3.84 (-2.72, 4.81), 1.00 \| 0.03 (-0.22, 0.35), 0.88 \| \| Exercise \| -3.81 (-2.70, 4.86), 1.00 \| -3.85 (-2.73, 4.81), 0.99 \| 0.05 (-0.14, 0.25), 0.97 \| \| Cardiovascular disease \| -3.82 (-2.92, 5.04), 0.93 \| -3.81 (-2.83, 4.93), 1.00 \| -0.01 (-0.65, 1.33), 0.97 \| \| DBP \| -3.61 (-2.66, 4.90), 0.97 \| -0.81 (-3.39, 5.24), 0.96 \| -2.80 (-2.24, 2.18), 0.96 \| \| TC \| -3.79 (-2.69, 4.88), 0.99 \| -3.54 (-2.69, 4.91), 0.99 \| -0.25 (-0.40, -.43), 0.95 \| \| LDL-C \| -3.80 (-2.69, 4.88), 0.99 \| -3.71 (-2.70, 4.88), 0.99 \| -0.10 (-0.22, 0.21), 0.99 \| \| HDL-C \| -3.87 (-2.70, 4.88), 0.99 \| -3.94 (-2.70, 4.86), 0.99 \| 0.07 (-0.12, 0.13), 1.00 \| \| Triglycerides \| -3.80 (-2.65, 4.91), 1.00 \| -3.99 (-2.78, 4.75), 0.99 \| 0.19 (-0.29, 0.54), 0.94 \| \| BMI \| -3.82 (-2.72, 4.85), 0.99 \| -4.01 (-2.80, 4.74), 0.99 \| 0.19 (-0.63, 0.59), 0.99 \| | | | |
| --- | --- | --- | --- | --- | --- | --- | --- | --- | --- | --- | --- | --- | --- | --- | --- | --- | --- | --- | --- | --- | --- | --- | --- | --- | --- | --- | --- | --- | --- | --- | --- | --- | --- | --- | --- | --- | --- | --- | --- | --- | --- | --- | --- | --- | --- | --- | --- | --- | --- | --- | --- | --- | --- | --- | --- | --- | --- | --- | --- |
| Results were reported as estimate (95%CI), p value.  Unadjusted values are reported. Abbreviations as in Table 1. | | | |
|  |  |  |  |
